# Uterine Intravenous Leiomyomatosis with Cardiac Extension: Radiologic Assessment with Surgical and Pathologic Correlation

**DOI:** 10.1155/2015/576743

**Published:** 2015-07-05

**Authors:** Go Nakai, Kazuya Maeda, Kazuhiro Yamamoto, Takashi Yamada, Yoshinobu Hirose, Yoshito Terai, Masahide Ohmichi, Takahiro Katsumata, Yoshifumi Narumi

**Affiliations:** ^1^Department of Radiology, Osaka Medical College, 2-7 Daigaku-machi, Takatsuki City, Osaka 569-8686, Japan; ^2^Department of Obstetrics and Gynecology, Osaka Medical College, 2-7 Daigaku-machi, Takatsuki City, Osaka 569-8686, Japan; ^3^Department of Pathology, Osaka Medical College, 2-7 Daigaku-machi, Takatsuki City, Osaka 569-8686, Japan; ^4^Department of Cardiovascular Surgery, Osaka Medical College, 2-7 Daigaku-machi, Takatsuki City, Osaka 569-8686, Japan

## Abstract

We present the computed tomography (CT) and magnetic resonance (MR) imaging findings of a 71-year-old woman with a cardiac extension of intravenous leiomyoma (IVL) that arose from the uterus, extended to the inferior vena cava (IVC), and reached the right ventricle through the right ovarian vein. Radiologic-pathologic correlation showed that the intravascular cord-like mass originating from the IVC and extending to the right ventricle was composed of degenerated smooth muscle cells with a number of large vessels that were regarded as arteries; moreover, the arteries within the cord-like mass appeared to be looping internally. Given the disappearance of the right ovarian venous wall around the IVL pathologically, extracting the tumor from the ovarian vein during an operation is considered to be impossible retrospectively. Also it was difficult to identify even the intravenous extension of the uterine leiomyoma histopathologically. Therefore, contrast-enhanced CT, in particular arterial phase imaging, provided important information that revealed the mass, range, and path of the lesion, ensuring that an appropriate operative plan could be drawn up and the tumor completely excised.

## 1. Introduction

Intravenous leiomyomatosis (IVL) is a rare neoplasm characterized by a smooth muscle cell tumor with a histologically benign appearance that grows within the uterine and pelvic venous system and inferior vena cava (IVC), even involving the heart. Although histologically benign, IVL can behave clinically in a malignant way, since sudden death can occur as a result of intracardiac leiomyomatosis with subsequent obstruction of venous return to the heart. Complete removal of the tumor is essential for the treatment of IVL. Therefore, precise information about the components of the uterine tumor and the complete path of extension of the lesion obtained with MRI and CT is required for surgical planning. However, no study has ever reported a detailed comparison between the imaging features of IVL and the histopathologic specimen. Here we present a case of intravenous leiomyoma that arose from the uterus, extended to the IVC, and reached the right ventricle through the right ovarian vein, including radiologic-pathologic correlation.

## 2. Case Report

A 71-year-old woman, gravida 6, para 4, with a two-week history of general malaise and no history of gynecological surgery, was referred to the department of cardiovascular medicine in our hospital since an echogenic mass in the right atrium was detected with cardiac ultrasonography. She had a history of hypertension, diabetes, and atrial fibrillation that had been well controlled by medication. Laboratory tests revealed normal results for tumor markers, liver and kidney function, and D-dimers, except for hemostatic abnormalities associated with anticoagulant drugs taken for atrial fibrillation.

**Figure 1:**
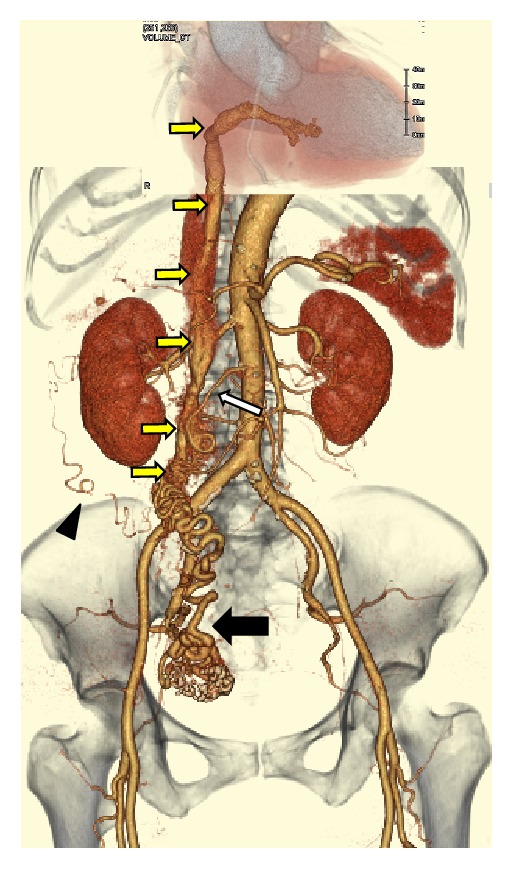
Three-dimensional arterial phase volume-rendered CT image indicated dilation of the right uterine artery (black arrow). Several small arteries originating from the right uterine artery were considered to be tumor arteries because they extended to the IVC through the right ovarian vein and then extended into the right atrium and the right ventricle (yellow arrows). A plexus of small arteries which comprised complex vascular formations originating from the right ovarian artery (white arrow), the right subcostal artery (arrowhead), and the right uterine artery surrounded tumor arteries along the route of the right ovarian vein.

**Figure 2:**
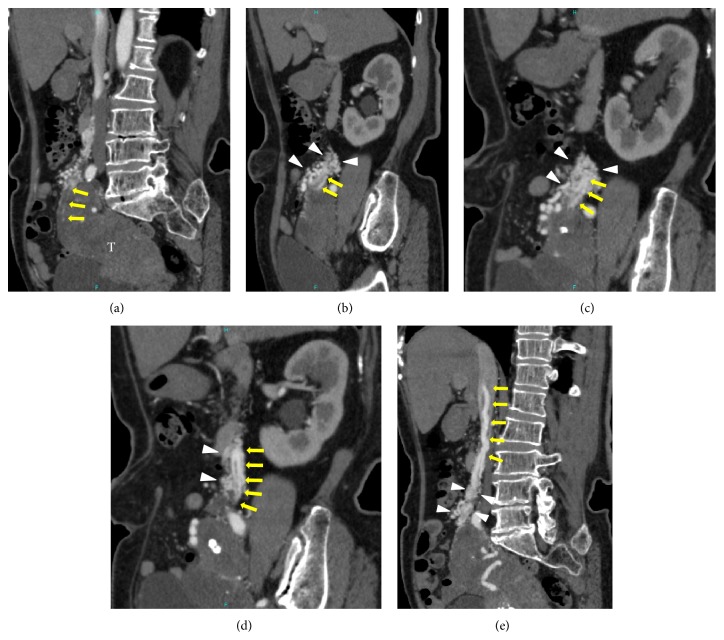
(a) Multiplanar reconstruction (MPR) images along the tumor arteries allowed for identification of the origin of the intravascular component (arrows) arising from the uterine tumor (T). ((b)–(e)) A plexus of small arteries, which comprised complex vascular formations (arrowheads) originating from the right ovarian artery, the right subcostal artery, and the right uterine artery, surrounded tumor arteries (arrows) along the route of the right ovarian vein and caused the diameter of tumor arteries to expand by communicating with each other at many locations.

Dynamic contrast-enhanced computed tomography (DCE-CT) of the chest and abdomen was performed to determine the extent of the tumor. Arterial phase contrast-enhanced CT (AC-CT) images indicated the presence of a hypodense uterine mass and dilation of the right uterine artery (RUA). Although it was difficult to trace the tumor arteries using only axial plane images, multiplanar reconstruction images along the tumor arteries enabled tracing of the tumor arteries through the route of the right ovarian vein. Several small arteries originating from the RUA were considered to be tumor arteries because they extended to the IVC through the right ovarian vein with increasing diameter and then extended into the right atrium and the right ventricle. A plexus of small arteries, which comprised complex vascular formations originating from the right ovarian artery, the subcostal artery, and the right uterine artery (Figure 1), surrounded tumor arteries along the route of the right ovarian vein and caused the diameter of tumor arteries to expand by communicating with each other at many locations (Figure 2).

**Figure 3:**
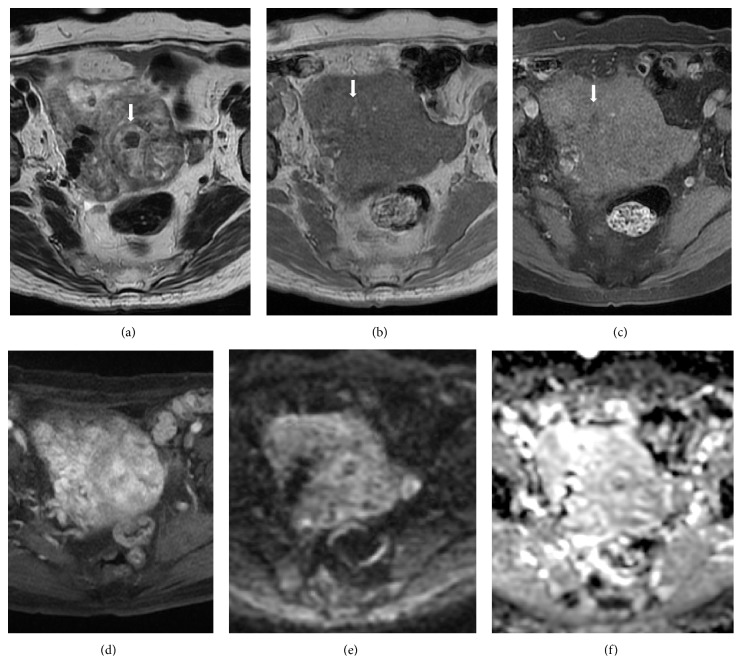
On MR images, the uterine tumor, approximately 13 × 7 × 6 cm in size, extensively involved the myometrium as a poorly demarcated diffuse lesion. (a) Axial T2-weighted image showed the lesion involving a few hypointense nodules (arrow) as having inhomogeneous high signal intensity (SI). (b) Axial T1-weighted image showed the lesion with a number of hyperintense tiny foci (arrow) as having low SI similar to that of the myometrium. (c) On the axial fat-suppressed T1-weighted image, the hyperintense foci within the lesion on T1-weighted images were suppressed (arrow), reflecting a lipomatous tumor. (d) On the contrast-enhanced fat-suppressed T1-weighted image, the lesion demonstrated strong contrast enhancement but contained several poorly demarcated hypointense areas, reflecting degenerative or necrotic changes. ((e), (f)) On diffusion-weighted images (e) and the ADC map (f), the lesion showed slightly high SI.

Magnetic resonance (MR) imaging was performed to assess the characteristics of the uterine tumor. On MR images, the uterine tumor, approximately 13 × 7 × 6 cm in size, extensively involved the myometrium as a poorly demarcated diffuse lesion. On T1-weighted images, the lesion exhibited low signal intensity (SI) similar to that of the myometrium and included a number of hyperintense tiny foci that were suppressed on fat-suppressed T1-weighted images, reflecting a lipomatous tumor. On T2-weighted images, the lesion exhibited inhomogeneous high SI and involved a few hypointense nodules. On contrast-enhanced fat-suppressed T1-weighted images, the lesion demonstrated strong contrast enhancement but contained several poorly demarcated hypointense areas, reflecting degenerative or necrotic changes. On diffusion-weighted images and the apparent diffusion coefficient (ADC) map, the lesion showed slightly high SI (Figure 3). On the basis of these findings, lipoleiomyoma was considered to be the most likely diagnosis for the uterine tumor.

**Figure 4:**
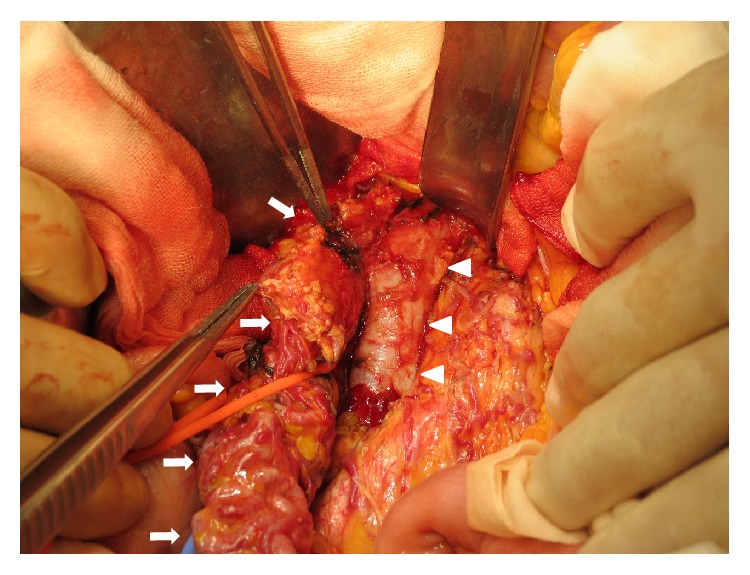
The right infundibulopelvic ligament including intravenous leiomyoma. The right infundibulopelvic (IP) ligament (arrows) including intravenous leiomyoma that arose from the uterus formed a connection to IVC (arrowheads) along the route of the right ovarian vein. A plexus of small arteries which were detected on arterial phase CT (Figures 1 and 2) was seen on the surface of the IP ligament.

The presumptive diagnosis of intravenous leiomyoma (IVL) was made based on a combination of CT and MR findings, and single-stage surgery with deep hypothermic circulatory arrest was scheduled. The operation was performed with a team of gynecologists and cardiac surgeons. First, a skin incision was made from the pelvic symphysis to the xiphoid process via the left side of the navel, and the surgical wound was separated by a retractor (Lobster “Wishbone”-style retractor system). The uterine fundus was enlarged with smooth surface. Bilateral adnexa and omentum were inspected and found to be normal with the exception of mild omental adhesion to the anterior abdominal wall. The right infundibulopelvic (IP) ligament had thickened to 3 cm in diameter since plexiform arteries arising from the right uterine artery surrounded the ligament. Furthermore, the plexiform arteries formed a connection to IVC along the route of the right ovarian vein (Figure 4).

**Figure 5:**
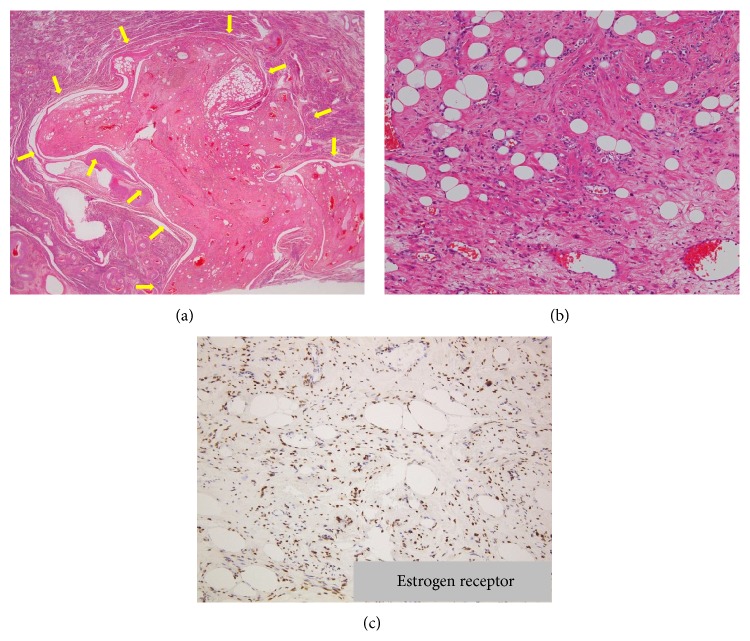
(a) HE staining showed the well-circumscribed uterine lesion protruding into the uterine myometrium (arrows). (b) The uterine lesion was composed of variable amounts of smooth muscle and fat cells with hydropic and hyaline degeneration, accompanied by numerous blood vessels. (c) On immunohistochemical staining, the nuclei of smooth muscle cells in the tumor were positive for estrogen receptor (ER) and progesterone receptor (PR) (not shown).

Secondly, a thoracotomy was performed, and a cardiopulmonary bypass was established with ascending aortic arterial return and venous drainage through the right atrial appendage. After the circulation was interrupted, systemic temperature was reduced to 22°C. The IVC was then opened below the renal veins. The intracardiac mass was found to be floating freely, with the exception of being slightly adhered to the internal surface of the right ventricle. While the cardiac surgeons separated the adhesion, the gynecologists incised the IVC via a venotomy and cut off the intracaval tumor at its origin. The intracardiac and intracaval components of the tumor were drawn out upward through the right atrial incision. The uterus was found to be enlarged with an intramural tumor. Moreover, a continuous plexus of small arteries was observed along the right ovarian vein from the uterine tumor to the IVC. Subsequently, transabdominal hysterectomy and bilateral salpingo-oophorectomy were performed, with the right ovarian vein surrounded by the plexus of small arteries removed as well. Through these procedures, complete tumor resection was achieved. She was discharged on the 18th postoperative day without any complications. Clinical and radiologic assessment over a 7-month follow-up has shown no evidence of tumor recurrence. Histopathologically, the uterine lesion was composed of variable amounts of smooth muscle and fat cells with hydropic and hyaline degeneration. An intravenous component was not identified in the resected specimen. The intravascular mass originating from the IVC and extending to the right ventricle was composed of degenerated smooth muscle cells with a number of large vessels. On immunohistochemical staining, the nuclei of smooth muscle cells in the tumor were positive for estrogen receptor (ER) and progesterone receptor (PR) (Figure 5).

## 3. Radiologic-Pathologic Correlation

**Figure 6:**
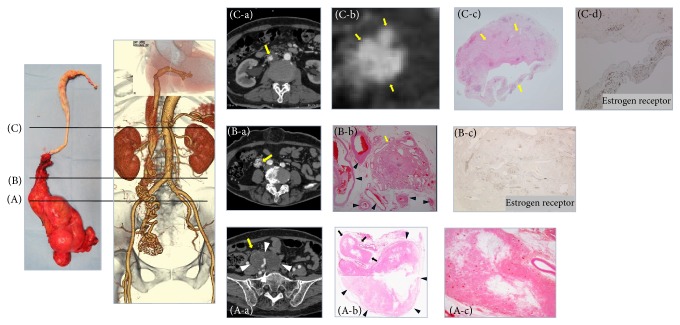
Radiologic-pathologic correlation. (A) The level of the origin of the extrauterine lesion. (A-a) AC-CT image revealed that the origin of the continuous mass extending into the IVC was a small nodule (arrow) on the surface of the uterine tumor (arrowheads); this was determined by referring to MPR images (Figure 2(a) arrows). (A-b) On a photomicrograph at the same level as the AC-CT image, the nodule could be identified as a small nodule of lipoleiomyoma (arrows) on the surface of the uterine lipoleiomyoma (arrowheads). (A-c) No vein wall structure was found around the nodule. (B) The level of the right ovarian vein. (B-a) AC-CT indicated the presence of plexiform arteries along the route of the right ovarian vein (arrow). (B-b) A photomicrograph at the same level showed a large number of vessels (arrowheads) surrounding a bundle of vessels with a small amount of smooth muscle cells (arrow). There was no vein wall structure around smooth muscle cells and hematoxylin-eosin (HE) staining showed no evidence of the tumor component. (B-c) The nuclei of smooth muscle cells stained positive for ER and PR (not shown), which indicated that they were a continuous part of the uterine tumor, but there was no evidence of an intravenous component. (C) The level of the IVC. (C-a) The intracardiac and intracaval components of the tumor appeared as a cord-like mass composed of just a bundle of arteries on the AC-CT image (arrow). (C-b) AC-CT image showed three arteries within the tumor (arrows). (C-c) Histologic examination revealed those components were composed of degenerated smooth muscle cells including several large vessels (arrows). (C-d) The smooth muscle cells stained positive for ER and PR (not shown).

Although the intravascular component around the uterine tumor could not be found histopathologically, AC-CT images revealed that the origin of the continuous mass extending into the IVC was a small nodule on the surface of the uterine tumor (Figure 6(A-a)). On a photomicrograph at the same level as the AC-CT image, the nodule could be identified as a small nodule of lipoleiomyoma on the surface of the uterine lipoleiomyoma (Figure 6(A-b)), but no vein wall structure was found around the nodule (Figure 6(A-c)). At the middle level of the right ovarian vein, AC-CT indicated the presence of plexiform arteries along the route of the right ovarian vein (Figure 6(B-a)). A photomicrograph at the same level showed a large number of vessels surrounding a bundle of vessels with a small amount of smooth muscle cells. There was no vein wall structure around smooth muscle cells and hematoxylin-eosin (HE) staining showed no evidence of the tumor component (Figure 6(B-b)). However, the nuclei of smooth muscle cells stained positive for ER and PR (Figure 6(B-c)). Thus, the bundle of vessels with a small amount of smooth muscle cells was considered to be a continuous part of the uterine tumor, but there was no evidence of an intravenous component. The intracardiac and intracaval components of the tumor appeared to be a cord-like mass composed of just a bundle of arteries on AC-CT images (Figures 6(C-a) and 6(C-b)). Histologic examination revealed that those components were composed of degenerated smooth muscle cells including several large vessels (Figure 6(C-c)). The smooth muscle cells stained positive for ER and PR (Figure 6(C-d)). The large vessels were assumed to be arteries because their number equaled the number of arteries on AC-CT images (Figures 6(C-b) and 6(C-c)). Moreover, these arteries appeared to loop within the intracaval and intracardiac components of the tumor as the end of the tumor did not appear to have clear arterial contact with the inner walls of the right ventricle during surgery as well as on AC-CT images.

## 4. Discussion

IVL is characterized by the presence of benign smooth muscle growth within the vascular spaces of venous and lymphatic spaces within the myometrium. Pathologic reports indicate that extrauterine involvement occurs in 30% to 80% of cases of IVL, and cardiac involvement occurs in 10% to 30% [1]. When the tumor propagates into the heart, it may extend to the right atrium (45.6% of cases), right ventricle (45.6%), or pulmonary vasculature (8.8%) [2]. To date, fewer than 300 cases have been reported in the English literature, with fewer than 100 of these cases having cardiac involvement.

The clinical characteristics of IVL mainly depend on the location and extent of the lesion. Even when the tumor extends intravenously as far as the IVC, patients may be asymptomatic until cardiac insufficiency develops due to intracardiac extension. More distal intravascular extension of the tumor can result in various cardiorespiratory symptoms such as shortness of breath, palpitation, and edema of the lower extremities.

Although the precise histogenesis remains unclear, two theories exist about the etiology of IVL [3, 4]. IVL is believed to arise from either intravascular projections of uterine myoma or directly from the wall of a uterine vessel. In the present case, we found that smooth muscle cells along the route of the right ovarian vein and those in the cord-like mass extending from the IVC to the right ventricle, as well as those in the main uterine tumor, stained positive for ER and PR. This may support the first theory, as endothelial and subendothelial cells express no to very weak ER and PR positivity. Furthermore chromosomal analyses have proposed a pathogenetic relationship between IVL and uterine leiomyoma with chromosomal transaction t(12;14) from the fact of the similarity in chromosomal rearrangements [5].

The differential diagnosis of IVL by imaging features mainly includes intravenous leiomyosarcomatosis and low-grade endometrial stromal sarcoma (ESS) with cardiovascular involvement. Intravenous leiomyosarcomatosis consists of proliferating malignant smooth muscle cells originating from the walls of the uterine or pelvic veins [3]. Typically, it is confined to the pelvis in the same way as IVL, but when exposed to cardiac venous blood flow, it may invade the venous wall or adjacent organs [6]. Furthermore, it is much rarer than IVL since very few cases have been documented in the literature so far [6–9]. In ESS, vascular space involvement has been reported in over 50% of surgical specimens, but further tumor extension to large vessels is rarely observed. Although several cases of ESS with cardiovascular involvement have been reported, all of these patients had a history of hysterectomy and their intravascular tumors arose from recurrent pelvic tumors that infiltrated adjacent organs such as the bladder and rectum. Such invasion is seldom seen in IVL [10]. In the present case, although the uterine tumor was depicted as a poorly demarcated diffuse lesion within the myometrium on MRI, a lipomatous component indicative of a benign lipomatous tumor such as a lipoleiomyoma [11–13], angiomyolipoma [14], or pure lipoma [15] was also detected within the tumor. Although liposarcoma, which consists of less-differentiated fat cells that have undergone sarcomatous change, can be included in the differential diagnosis, it is exceptionally rare, with only 13 cases having been reported in conjunction with intravenous leiomyosarcomatosis [16]. In this respect, MRI may be able to provide precise information about the characteristics of the uterine tumor.

With regard to the path of extension, AC-CT images demonstrated several small arteries emerging from the uterine tumor and extending to the IVC and the right heart via the right ovarian vein. However, HE staining did not show intravenous components composed of smooth muscle cells along the route of the right ovarian vein as no venous wall was confirmed around the plexiform arteries with a small amount of smooth muscle cells. It was therefore histopathologically impossible to prove intravenous extension of the uterine tumor based on anything other than the cord-like tumor extending from the IVC to the right ventricle. Thus, difficulty in identifying the intravenous extension of a uterine leiomyoma can result in misdiagnosis of the tumor histopathologically, leading to inadequate patient follow-up after hysterectomy and regrowth of residual intravenous tumor tissue. We assume that this is one of the reasons why IVL is usually diagnosed after an operation for uterine myoma. In actuality, 64% of women diagnosed with IVL had undergone a previous hysterectomy, with a range of 6 months to 20 years before presentation with the intravenous portion of the tumor [17].

The cord-like mass extending from the IVC to the right ventricle was histopathologically composed of degenerated smooth muscle cells including plexiform vessels. When the number of vessels within the cord-like mass was compared to the number on AC-CT images at the same level, all vessels were regarded as arteries because the same number of arteries was detected on AC-CT images. However, it is a point of note that the cord-like mass only had feeding arteries without any drainage veins and, moreover, that the end of the tumor was not found to have clear arterial contact with the inner walls of the right ventricle during surgery as well as on AC-CT images. These results suggest that the arteries within the cord-like mass were looping internally.

Complete removal of the tumor is the standard therapy for IVL to prevent recurrence. Given the disappearance of the right ovarian venous wall around the IVL pathologically, extracting the tumor from the ovarian vein during an operation is considered to be at risk of bleeding as well as impossible. Therefore, the removal of the tumor separately by cutting off the intracaval tumor at its origin was assumed to be a safe way to accomplish complete resection of the tumor. In premenopausal patients with IVL, a bilateral oophorectomy should also be performed since estrogen would stimulate the tumor to grow.

In conclusion, IVL has some distinctive features on imaging. AC-CT should provide precise information to reveal the mass, the extent, and the complete path of the lesion, ensuring that an appropriate operative plan is created and that the tumor is completely excised. Furthermore, MRI can give additional detailed information on the components of the uterine tumor.
